# Where healthcare systems fall short in long-term ostomy care: implications for quality of life, healthcare utilization, and sustainable care pathways

**DOI:** 10.3389/fgstr.2026.1920507

**Published:** 2026-08-27

**Authors:** Judith A. Hargreaves, Diego Schaps, Beata Buchelt

**Affiliations:** 1 Global Director of Market Access, Ostomy Care, ConvaTec, Paddington, London, United Kingdom; 2 Department of Surgery, Duke University Health System, Durham, NC, United States; 3 Department of Human Capital Management, Krakow University of Economics, Lesser Poland, Krakow, Poland

**Keywords:** healthcare cost, ostomy, patient care pathways, quality of life, self-management, sustainable development

## Abstract

**Introduction:**

Ostomy surgery, most commonly following colorectal cancer, can create a lifelong self-management burden affecting physical health, psychological well-being, and daily functioning. This narrative review synthesizes evidence on long-term ostomy self-management, its multidimensional burden, and interventions and care pathway designs that support sustainable patient-centered outcomes.

**Methods:**

This narrative review was informed by a structured literature search across PubMed/Medline, Embase, Cochrane Library, CINAHL, ClinicalTrials.gov, ICTRP, and Google Scholar (2020–2026), supplemented by ostomy organization resources and some foundational pre-2020 studies. Literature considered for inclusion were observational, cohort, cross-sectional, qualitative and mixed-methods studies, systematic reviews, meta-analyses, and registry analyses.

**Results:**

92 primary studies met the eligibility criteria. Nine thematic domains were identified: self-management burden, quality of life (QoL), healthcare utilization, intervention effectiveness, predictive factors, long-term adaptation, lived experience, economic burden, and environmental sustainability. Ostomy complication rates are high: peristomal skin lesions affect 52–75% of patients, and leakage is reported by 27–58%. Structured education pathways increased independent self-care from 8% to 68% at discharge and reduced home nursing reliance from 80% to 50%. Telehealth and continuing care interventions reduced 30-day readmissions from 29% to 14%. Ostomy-related complications are associated with a substantial, long-term economic burden encompassing both direct (hospitalization and outpatient services) and indirect costs (productivity losses). Body image concerns and sexual dysfunction persist among long-term survivors, and real lived experience research identifies continuous cognitive, emotional, and practical burdens that clinical metrics alone do not capture. Environmental sustainability is an emerging dimension of ostomy care, and ostomy-specific evidence remains limited.

**Conclusion:**

Longitudinal, patient-centered care pathways integrating education, specialist follow-up, psychosocial support, and digital tools have been associated with improved self-management and QoL and with reductions in complications and unplanned healthcare use; these approaches may also help lower the economic and environmental burden of long-term ostomy care.

## Introduction

Approximately 13.5 million people worldwide are living with an ostomy, most commonly following colorectal cancer (CRC) (1). CRC remains the leading cause of permanent ostomy formation, with incidence increasing in both older and younger adult populations (2). Other indications include inflammatory bowel disease, diverticulitis, and spinal cord injury, making ostomy creation a life-saving intervention across a broad patient population (3).

For many patients, living with an ostomy imposes a substantial and often lifelong self-management burden encompassing physical, psychological, and social challenges, although the nature and severity of this burden vary considerably across individuals and ostomy trajectories (3–6). Patients frequently experience persistent impairments in quality of life (QoL) (7) and high complication rates (7–9). These long-term challenges underscore the need for structured, longitudinal care that extends well beyond surgery, integrating specialist nursing, patient education, and psychosocial support to enable effective self-management across the full duration of survivorship (10, 11). Existing evidence shows that self-efficacy, access to specialist care, and structured support programs can mitigate ostomy-related patient burden (12–15). Nonetheless, many unmet needs remain, such as limited culturally tailored resources, insufficient digital or remote support tools (10, 16), inadequate caregiver and family support (17), psychosocial gaps, and poor continuity of care (18, 19).

International and regional ostomy organizations [International and regional organizations, including the International Ostomy Association (IOA), European Ostomy Association (EOA), Ostomy Association of the Americas (OAA), Asian and South Pacific Ostomy Association (ASPOA)] and national bodies such as the United Ostomy Associations of America (UOAA) promote high standards of pre- and postoperative education and accessible patient resources (20–24). When these efforts are embedded within structured, patient-centered care pathways that integrate education and sustained follow-up, outcomes have been associated with greater patient independence, lower readmission rates, and more efficient use of healthcare resources (11, 25, 26).

Despite this growing evidence base, research and quality improvement efforts in ostomy self-management have often proceeded in isolation, with limited synthesis of what has been attempted, what has worked, and where evidence gaps persist (9, 27–30). Consolidating this evidence base may help reduce duplication of effort and better direct resources toward interventions with demonstrated benefit. This review addresses that gap directly. By synthesizing the current evidence across the full scope of ostomy self-management – from clinical burden and QoL to healthcare utilization, economic impact, and environmental sustainability – it provides a consolidated evidence base that clinicians, researchers, and quality improvement professionals can use to design, refine, and build on existing approaches rather than starting from scratch. The aim is to unify the field around what the evidence shows works, and to identify where the most important unanswered questions remain.

## Literature search

This is a narrative review of the global evidence on the long-term burden of ostomy self-management and its impact on QoL and healthcare utilization. The review was informed by a structured literature search rather than free-text browsing: a comprehensive search was performed across PubMed/Medline, Embase, Cochrane Library, CINAHL, ClinicalTrials.gov, the International Clinical Trials Registry Platform, and Google Scholar, supplemented by resources from relevant international ostomy organizations, including the IOA, EOA, OAA, ASPOA, and UOAA (full database-specific search strings and article selection flow are provided in **Supplementary Tables 1**, **2**. Consistent with a narrative rather than systematic design, this review does not follow the Preferred Reporting Items for Systematic Reviews and Meta-Analyses (PRISMA) reporting standard and does not apply formal risk-of-bias or Grading of Recommendations Assessment, Development and Evaluation (GRADE) appraisal; findings should be interpreted as a qualitative, thematic synthesis of the literature rather than a quality-weighted, pooled estimate.

English-language studies from 2020 to 2026 were included. A small number of earlier (pre-2020) studies were additionally cited where foundational to a specific clinical concept discussed in the review (e.g., original validation of an outcome measure or a landmark clinical trial) and not superseded by more recent evidence; these were identified through the authors’ therapeutic area expertise rather than through the systematic search. The eligible population was defined *a priori* as adults living with a permanent or temporary ostomy, primarily due to CRC, inflammatory bowel disease, or diverticular disease, and related conditions requiring ostomy formation. Included studies examined self-managed ostomy care and related interventions, encompassing structured education, psychosocial support, continuity of care, digital and telehealth tools, culturally tailored resources, and caregiver involvement. Eligible study designs included original research (observational, cohort, cross-sectional, qualitative, and mixed-methods studies), systematic reviews, meta-analyses, and registry analyses. Excluded studies were those focused exclusively on pediatric populations, short-term perioperative outcomes without longer-term follow-up, or non-peer-reviewed commentaries and case reports without systematic data.

Sources identified through the database and organizational searches were screened for relevance against the criteria above by the lead author and cross-checked by co-authors. Inclusion criteria, thematic domains, and evidence interpretation were agreed and cross-checked among all three authors. Thematic domains were defined *a priori* by the authors from the recurring outcome categories reported in the ostomy self-management literature and from domains emphasized by international ostomy organizations. Data were extracted against these domains and synthesized narratively – an inductive, pattern-based approach appropriate to a heterogeneous evidence base spanning quantitative, qualitative, and mixed-methods sources – to identify consistent findings, points of divergence, and evidence gaps within and across domains.

## Findings

A total of 92 primary studies, together with foundational and organizational sources, informed the synthesis. Findings are organized across nine domains reflecting the recurring outcome categories in the ostomy self-management literature and the cross-cutting themes emphasized by international ostomy organizations: self-management burden and challenges; quality of life; healthcare and specialist service utilization; effectiveness of support interventions; the role of specialist ostomy nursing; predictive factors; long-term trajectories and adaptation; real lived experience; economic burden; and environmental sustainability. These domains progress from the clinical and experiential burden of ostomy self-management toward the interventions, predictors, and system-level implications that follow from it.

### Self-management burden and challenges

Ostomy self-management can impose a substantial and enduring burden on patients, particularly those living with long-term CRC (27, 31, 32) (**Table 1**). This burden spans technical, physical, and practical dimensions of daily care, with patients frequently reporting leakage, skin complications, pain, and the need for frequent pouch changes that can add more than 30 minutes of care time per day (27, 31).

**Table 1:** Prevalence of common challenges in ostomy self-management.

Domain	Key challenges	Prevalence
Technical	leakage	27–58% (16, 27, 31)
	postoperative hernia	27% (27)
	frequent pouch changes	26% (27)
	stomal retraction (>0.5 cm below skin surface)	14% (37, 38)
	stomal prolapse (colostomy)	10% (37, 39)
Physical	skin complications (general)	2.9–81.1% (31, 40)
	peristomal lesions	52–75% (10, 33)
	troublesome odor	55% (31)
	pain	21–55% (31, 34)
	mobility issues	32% (34)
	multiple concurrent ostomy challenges	31% (27)
	restricted daily activities	19–29% (31, 34)
	skin excoriation	23.3% (32)
	parastomal hernia	18.6% (32)
	prolapse	18.6% (32)
Psychological	anxiety/depression	13–31% (18)
Device/adjustment	self-sufficiency at 1 year	60–90% (10, 35)
	dissatisfaction with the device	38% (10)
Self-care burden	at least one self-care problem	63% (27)
	two or more self-care problems	31% (27)

Physical complications are common and contribute heavily to this burden. Overall, 51.2% of patients experience at least one complication, with 18.6% reporting two or more (32). Peristomal skin lesions are observed in approximately half of patients at postoperative assessment and in up to three-quarters following discharge (10, 33). Specific complications include skin excoriation, parastomal hernia, and stomal prolapse (32). Troublesome odors and pain are reported in up to 55% of patients, and mobility difficulties by approximately 32% (31, 34). The prevalence of common challenges across technical, physical, psychological, and appliance domains is summarized in **Table 1**.

Self-care ability and autonomy vary widely across patients. One year post-surgery, between 60% and 90% of patients achieve self-sufficiency in ostomy care (10, 35), yet 38% report dissatisfaction with their ostomy device (10). Insufficient product support further increases the time burden and reduces confidence in self-management (16).

Among long-term CRC survivors, over half report at least one ostomy-related self-care challenge, and nearly one-third experience multiple concurrent difficulties (27). Qualitative studies highlight the pervasive impact of ostomy management on daily life, including restrictions on clothing and diet, concerns about gas and odor, difficulties with bowel elimination, and disruption of sleep (36).

### QoL and domain-specific impacts

**Figure 1:**
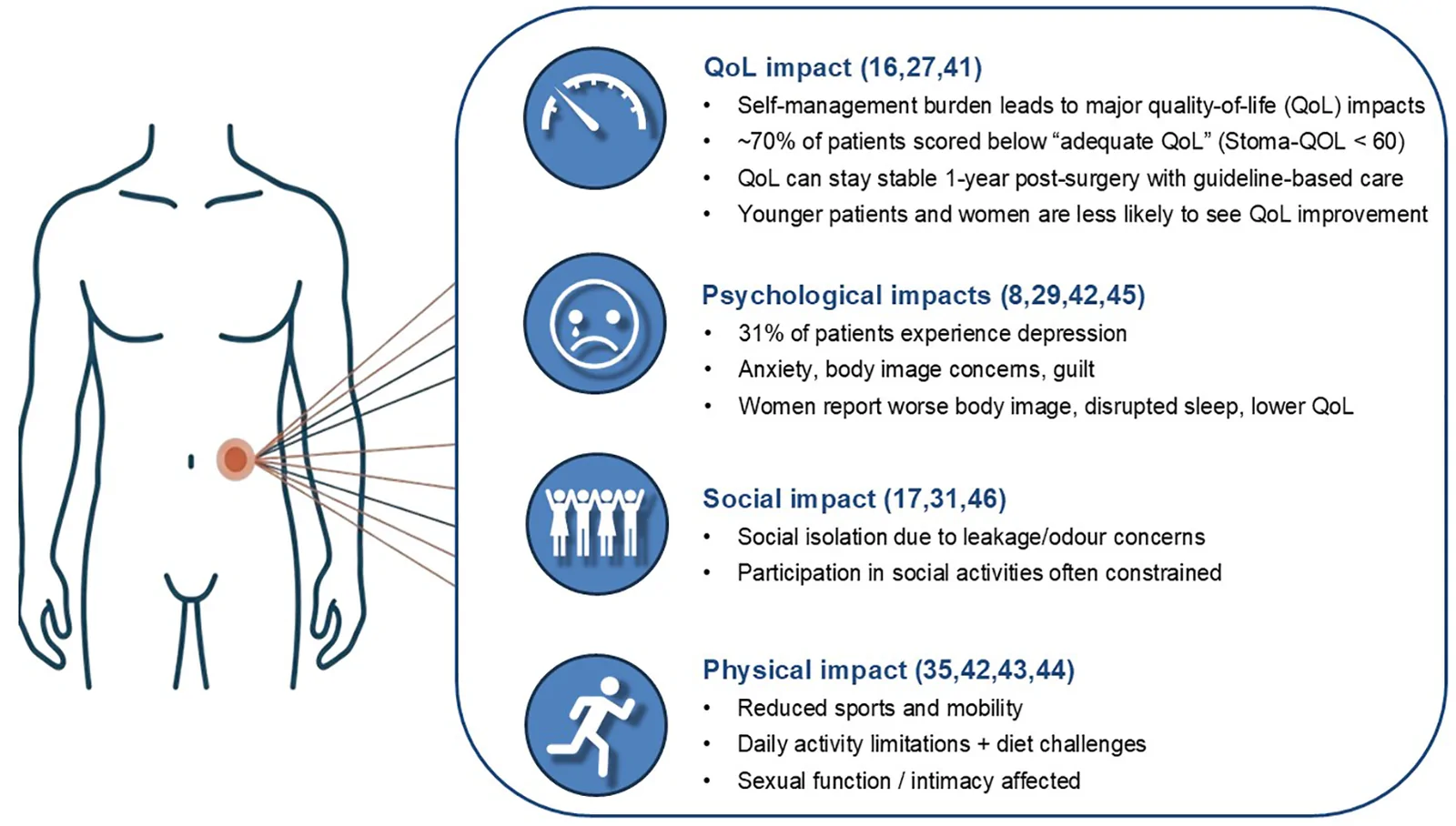
The burden of self-management translates directly into multidimensional quality of life impairments.

The burden of self-management translates directly into multidimensional QoL impairments across physical, psychological, and social domains (16, 27, 41) (**Figure 1**). Across European and Asian cohorts, median scores on instruments such as Stoma-QOL and the Short Form-36 indicate substantial limitations, with approximately 70% of patients scoring below thresholds for adequate QoL (Stoma-QOL <60) (32, 35). Patients managed according to evidence-based guidelines generally maintain stable QoL in the first year post-surgery, although younger patients and women are less likely to show improvement over this period (30).

Physical impacts include reduced participation in sports, limited mobility, challenges in daily activities, diet management difficulties, and compromised intimacy (35, 42–44). Psychological effects are prevalent, with 31% of patients experiencing depression, alongside anxiety and guilt (18, 29, 45). Women report greater challenges with body image, sleep disruption, and overall lower QoL compared with men (42). Social participation is frequently constrained by concerns about leakage or odor, with sexual function particularly affected (46).

Evidence indicates that targeted interventions can improve QoL across these domains. Exercise programs (47), preoperative stoma site marking (48), structured education (45, 49), family and social support (17), and mental health support (6, 50) have each demonstrated benefit. Nevertheless, QoL deficits frequently persist over the long term, emphasizing the need for sustained, patient-centered strategies rather than time-limited interventions (27, 34, 36).

### Healthcare and specialist service utilization

Healthcare utilization outcomes are less consistently reported in the ostomy literature than QoL, but available evidence indicates that structured support improves care efficiency and reduces unnecessary resource use. Early hospital discharge, defined as 1–2 days post-surgery, does not increase 30-day readmission rates (51), suggesting that well-supported early discharge is safe when accompanied by adequate follow-up. Continuity-of-care interventions, including standardized rehydration protocols and patient education, have reduced 30-day ileostomy readmission rates from 29% to 14% in quality improvement studies (52). Structured post-discharge support programs spanning 1–18 months have been associated with reductions in readmissions (odds ratio 0.45) and emergency visits (odds ratio 0.37) (53). Nurse-led discharge planning and patient education reduce unplanned outpatient visits without increasing overall care costs (17, 54).

Access to specialist ostomy services varies considerably across health systems. Regular follow-up at 3, 6, and 12 months post-surgery is reported in Scandinavian settings (35), but significant gaps exist elsewhere. In one Italian nationwide survey of 403 patients, 76% had not attended preoperative training and only 47% received professional care following discharge, yet 60% were self-sufficient in ostomy management at the time of assessment (10). Telehealth interventions have been shown to reduce hospital visits and consultation time, though some studies report a modestly higher likelihood of accessory costs among telehealth users compared with those receiving in-person care (p = 0.063) (37, 55). Collectively, these findings indicate that structured, accessible care pathways have the potential to reduce unnecessary healthcare utilization while maintaining or improving patient outcomes (10, 51, 54). At a time when specialist nursing and allied health workforces are under sustained pressure globally, this represents more than an efficiency gain: care models that build genuine patient self-sufficiency reduce unplanned clinical contact, freeing specialist capacity for patients with the greatest complexity and need (56, 57).

### Effectiveness of support interventions

Structured education and training have been consistently associated with improved self-management, independence, QoL, and clinical outcomes, and with fewer complications and readmissions (17, 49, 54, 58). A 4-day in-hospital educational pathway increased the proportion of patients achieving independent stoma care at discharge from 8% to 68%, and reduced reliance on home nursing services from 80% to 50% (58). Preoperative stoma site marking alone reduced complication rates by approximately 53%, improved self-care ability, and enhanced health-related QoL (48). Clinical guidelines from the American Society of Colon and Rectal Surgeons reinforce the importance of preoperative site marking, perioperative education, and structured care pathways for reducing morbidity, optimizing postoperative recovery, lowering readmission rates, and improving long-term outcomes (59). Integration of these components within Enhanced Recovery After Surgery programs further shortens hospital stay without increasing complication rates (45, 60).

Digital and telehealth interventions represent an expanding evidence base for improving self-management and psychosocial adjustment (16, 61–66). Multimedia messaging programs, app-based educational tools, and digital leakage notification systems have demonstrated improvements in self-efficacy, Patient Activation Measure scores, and WHO-5 Well-Being Index outcomes (16, 61–66). A systematic review and meta-analysis of telehealth stoma care interventions reported improvements in stoma adjustment (standardized mean difference [SMD] 1.44) and self-efficacy (SMD 10.23), although effects on anxiety and broader QoL measures were more variable across studies (66).

Peer support and psychosocial interventions also play an important role. Programs including Ostomy Self-Management Training, mindfulness-based exercises, the STOMA psychosocial intervention program, and StomieCare have each demonstrated improvements in stoma acceptance, coping, confidence, and psychosocial adaptation (18, 67–70). Even a single early peer visit in the postoperative period can provide meaningful benefit to new ostomates (70). Caregiver-focused interventions improve both patient engagement and caregiver well-being (67).

Despite these advances, many patients continue to lack access to sustained long-term follow-up, facing barriers that include geographical distance, cost, transport limitations, alongside constrained specialist nursing access discussed further below (10, 17, 18, 71). These findings highlight the need for individualized, multi-component interventions that target the key nodes of psychosocial adaptation at each stage of the patient journey (72, 73).

### The role of specialist ostomy nursing

Specialist ostomy nursing is a recurring predictor of good outcomes across the evidence reviewed above, including the benefits of nurse-led discharge planning already noted in the healthcare and specialist service utilization section (17, 54) and the association between regular specialist follow-up at 3, 6, and 12 months and better long-term adjustment in health systems where it is consistently available (35). Conversely, limited access to certified ostomy nurses is a recurring barrier to self-sufficiency and psychosocial adaptation. In one Italian national survey, only 47% of patients received professional nursing care following discharge, although 60% were nonetheless self-sufficient in ostomy management at the time of assessment (10). Broader gaps in preoperative training, partner and spousal support, and social reintegration support have each been linked to insufficient specialist nursing access (17, 18, 71). Wound, ostomy, and continence (WOC) nurses play a central role in optimizing pouching, troubleshooting complications, and supporting patient adaptation, yet access to this expertise remains inconsistent across health systems (74). This evidence is considered alongside the broader nursing workforce pressures discussed later in this review, which constrain the scalability of nurse-led models even where their clinical and cost-effectiveness is well established.

### Predictive factors for outcomes

Outcomes in ostomy self-management are influenced by a range of demographic, clinical, psychosocial, and system-level factors, as summarized in **Table 2**. With regard to patient-level factors, younger age is associated with more self-care challenges, while older age combined with inadequate education and limited home care support also increases difficulty (27, 75). Female sex and higher body mass index are associated with greater complication risk (27, 75). Ostomy type and permanence influence adjustment: colostomy patients generally adjust more readily than those with ileostomy, and permanent ostomies are associated with higher long-term adjustment scores compared with temporary ostomies (76, 77). Comorbidities, including diabetes, smoking, and chronic kidney disease, further increase the risk of complications and poorer outcomes (78).

**Table 2:** Predictive factors in ostomy self-management and outcomes (clinical and patient-reported).

Domain	Factors improving outcomes	Factors worsening outcomes
Patient-related	higher self-efficacy (66, 76, 79); adequate social/caregiver support (17); health literacy (81, 82); psychological well-being (79, 83); effective self-management (84, 85)	younger age (27, 30); older age (75); female sex (30, 42); higher BMI (27); anxiety/depression/guilt (18, 29, 34, 46); limited social/caregiver support (80); clothing restrictions/stoma site (36); time burden (16); poor self-management (71); longer time to learn self-management (32); comorbidities (diabetes, smoking, chronic kidney disease) (78)
Clinical/ostomy-related	colostomy rather than ileostomy (35); permanent ostomy rather than temporary (76)	ileostomy (35); postoperative complications (leakage, peristomal skin disease) (76); high-output stoma (27); frequent appliance changes (27); device adherence or allergy (36)
Care pathway/system	structured pre- and postoperative education (17, 49, 60); regular specialist follow-up (35, 37); discharge planning and continuity of care (54, 75); preventive care models (35); socio-educational interventions (86); self-sufficiency (10, 35)	lack of structured education (10, 75); limited access to specialist ostomy nurses (18, 71); fragmented hospital-community transitions (75); lack of long-term support (71)
Psychosocial/behavioral	effective coping (79, 87); engagement in self-care (15, 79); peer support (18); mental health screening (83)	impaired body image (34); intimacy challenges (42–44); lack of psychological support (10)
Technology/support	digital/remote support tools (37, 61–65); digital leakage detection (16)	

BMI, body mass index.

Modifiable factors represent the strongest targets for intervention. Preoperative stoma site marking, structured education, and ongoing specialist support are consistently associated with improved self-care, reduced complications, and enhanced QoL (45, 48, 59). Psychosocial factors, particularly self-efficacy, are key predictors of adjustment and health-related QoL (79). Structural equation modeling has demonstrated that both self-care maintenance and self-monitoring independently and positively predict QoL outcomes (79). Complications in turn independently predict poorer outcomes and greater ongoing support needs (76, 80), reinforcing the importance of early identification and proactive management of at-risk patients.

### Long-term trajectories and adaptation

**Figure 2:**
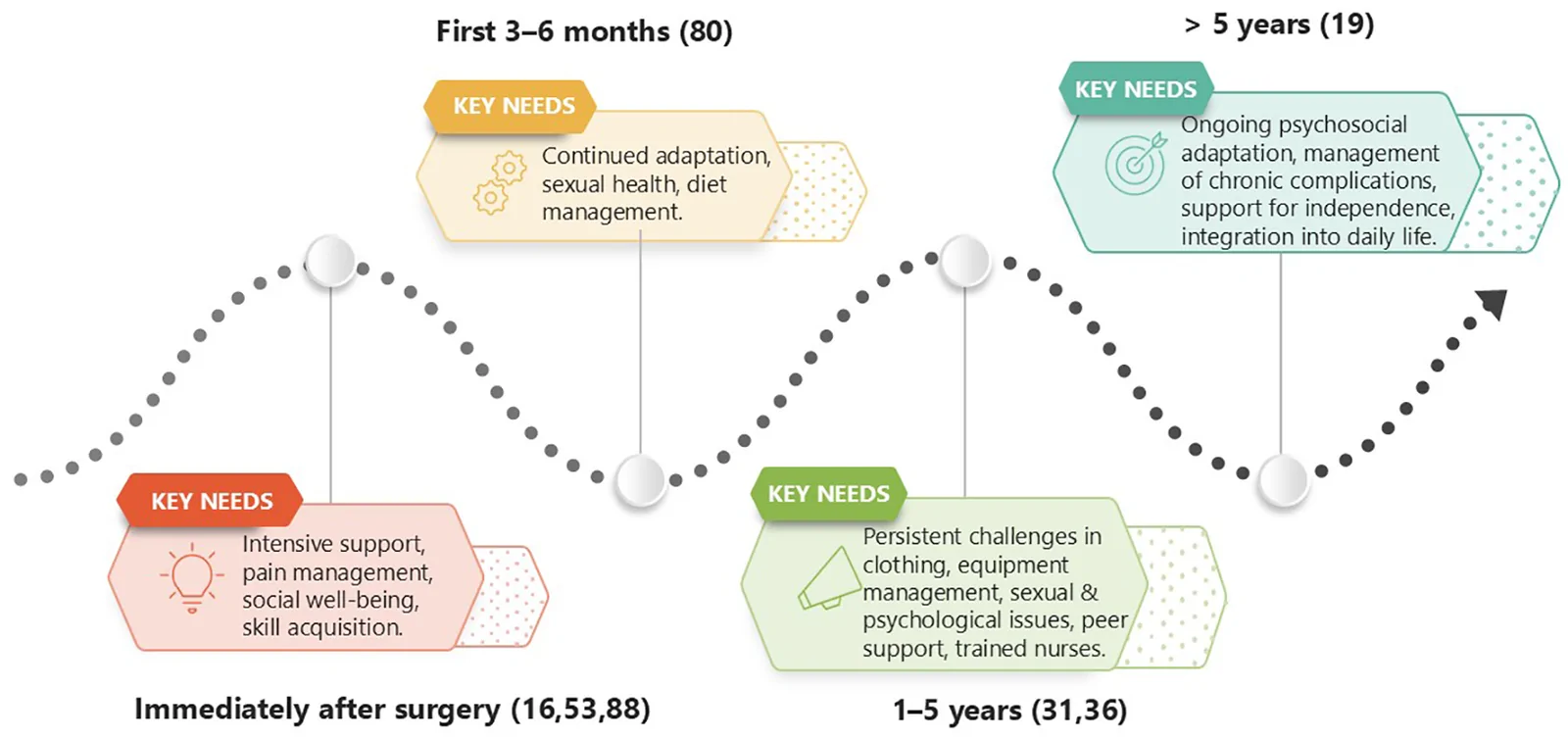
Long-term adaptation and support needs.

Supportive care needs are most critical in the immediate postoperative period and during the first year following surgery, with the first and third postoperative months identified as periods of particularly high need (16, 53, 88) (**Figure 2**). Needs generally decrease over the first 6 months, although sexual health concerns tend to increase over this period (80). Structured interventions during this critical early phase improve self-management, reduce anxiety, and enhance QoL (16, 88, 89).

Among long-term survivors of more than 5 years, persistent challenges remain common. These include clothing and dietary restrictions, ongoing equipment management difficulties, sexual and psychological challenges, and continued reliance on specialist nursing and patient association support (31, 36). Long-term colostomy patients can achieve high levels of autonomy and acceptance over time, yet specialist nursing support and access to patient organizations remain important across the full duration of survivorship (19).

Registry-level evidence reinforces the scale of the long-term burden. A nationwide Swedish registry study of 40,988 patients confirmed that ostomy surgery creates a high and sustained disease burden on healthcare systems for at least a decade, driven primarily by hospitalizations and complication-related care (28). Prehabilitation programs and structured education pathways have been shown to improve independence, reduce anxiety and depression, and enhance QoL, underscoring the value of longitudinal, patient-centered approaches to achieving sustainable self-management over the long term (44, 73) (**Figure 2**).

### Real lived experience

Real lived experience research highlights that ostomy management extends well beyond clinical outcomes, encompassing continuous cognitive, emotional, and practical demands. Patients can experience psychological burdens including altered self-image, persistent insecurity, lifestyle restrictions, and social detachment through self-stigmatization and identity loss (90). Distress related to embarrassment from leaks, odors, or noise leads many to fear public situations and feel anxious about returning to work and maintaining relationships (91, 92). Stoma creation is associated with reduced autonomy, decreased social interaction, and declining self-esteem, affecting QoL even in clinically stable patients (87). Peristomal skin complications in particular have been linked to reduced social interactivity and a loss of confidence in social and family relationships and in patients’ ability to resume normal daily activities (33).

Adaptation is not linear. Patients oscillate between periods of adjustment and renewed challenge, particularly during transitions such as returning to work, traveling, or changes in health status, with return to work conceptualized as a critical milestone in resuming family and social life (92). These findings reinforce the need for care pathways that provide support at points of transition, not only in the immediate postoperative period (93).

### Cost and economic burden

Ostomy care imposes a substantial and sustained economic burden encompassing surgical costs, long-term follow-up, complication management, and ongoing appliance expenditure. Hospitalization and outpatient services are the primary cost drivers, with complication-related care contributing disproportionately to total expenditure (28). A Swedish registry study of 40,988 patients reported elevated annual healthcare costs for at least a decade post-surgery, with 19–22% of 30-day readmissions ostomy related (28), while longitudinal Danish cohort data confirm that costs are front-loaded in the first 2 years, substantially exceeding matched controls (94).

Costs are substantial across systems: 6-month care costs in the United States (US) range from $18,000 to $80,000 (95, 96), and new colostomy healthcare resource utilization costs exceed £3,200 per patient in England (97). In 2024, peristomal skin complications alone cost approximately £349 per episode and up to £28.1 million annually across England (98, 99). In the US, out-of-pocket expenses commonly range from $1,100 to $2,700 (US dollars as of 2020) annually, and can include pouches, barrier rings, belts, adhesive removers, ostomy deodorant, and skin protective wipes, compounding inequities in access (100). Poor self-care amplifies all of these costs within an expanding global market (27, 28, 94, 101).

Effective interventions reduce this burden. Digital self-management interventions improve self-care (SMD 0.85) and QoL (SMD 0.64) (14), advanced practice nurse models in Spain are cost-effective at approximately €2,297 per quality of life year gained (as of 2020) (102), and continuing care interventions reduce complication rates (risk ratio [RR] 0.71), though effects on utilization and total costs require further prospective study (103). US telehealth program costs of approximately $1,758 (2018 USD) per patient provide a basis for modeling system-level savings (104).

### Environmental sustainability

Environmental sustainability is an emerging and, to date, comparatively under-researched dimension of ostomy care, given its reliance on single-use products and continuous medical waste generation. Contemporary pouches incorporate elaborate synthetic polymer assemblies that represent a significant departure from the more sustainable reusable rubber designs they replaced, and despite seven decades of use, stoma pouch design has not advanced substantially in terms of material sustainability (105). Opportunities exist for biodegradable pouches, composite materials, and personalized 3D-printed systems that better align clinical performance with environmental responsibility (105). Direct, ostomy-specific life-cycle data remain scarce; the evidence is drawn largely from broader healthcare sustainability research and should be interpreted as indicative of the scale of the issue rather than as ostomy-specific quantification.

Disposable consumables contribute to plastic waste across the full life cycle of production, distribution, and disposal, with hospital waste systems typically directing all medical device waste to incineration at high environmental and financial cost (106). Healthcare accounts for approximately 4.4% of global net carbon emissions (107). Direct CO_2_ emissions from fuel use in healthcare – primarily gas used for hot water – accounted for 10% of total emissions, while indirect CO_2_ emissions associated with purchasing goods and services from other sectors made up nearly 90% (108). Supply chain and care delivery emissions account for approximately 71% of the sector’s worldwide footprint, meaning energy transition alone addresses only the remaining 29% (109). Meaningful decarbonization requires action at the level of procurement, product life cycle design, and care pathway configuration (109, 110). For ostomy care, improvements in appliance reliability that reduce unplanned changes and pathway redesigns that reduce unnecessary hospital contact both represent practical contributions to reducing a long-term environmental burden.

## Discussion

Across the nine domains reviewed, the most consistent findings are that physical complications – particularly peristomal skin lesions and leakage – remain common; that structured education and continuity-of-care interventions produce the largest and most consistent gains in self-sufficiency and reductions in readmissions; and that QoL, body image, and economic burdens persist over the long term even when acute complications are well controlled. The evidence synthesized in this review indicates that ostomy management can impose a substantial, enduring burden extending across physical, psychological, social, economic, and environmental dimensions.

Taken together, the findings of this review point to a structural imbalance in how long-term ostomy care is organized: pathways are frequently built around managing the stoma and its complications, and patients are then supported to cope with the consequences of that management, rather than being supported from the outset to live full lives in which the ostomy is one manageable part. Reorienting pathways so that the person, not the ostomy, is the starting point for care design is a thread that connects each of the domains examined below.

While self-management is central to sustainable chronic care, it cannot be achieved without structured, longitudinal support. A 2022 systematic review demonstrated that care pathways incorporating pre- and postoperative education, discharge planning, and follow-up support consistently improved patient satisfaction and reduced both hospital length of stay and readmission rates (26).

The findings highlight a persistent tension between the critical importance of the early postoperative period and the limitations of care as it is currently delivered. This phase is pivotal for establishing self-management skills, preventing complications, and supporting psychological adaptation (37, 79, 80), yet evidence consistently identifies barriers across all perioperative phases, including inadequate preoperative preparation, in-hospital challenges with appliance selection, and post-discharge gaps in supply access, skin care support, and community follow-up (36, 49, 66, 81, 111, 112). These structural deficiencies help explain why interventions with well-established clinical and cost effectiveness – including specialist nurse models and digital self-management tools – have not translated into routine practice at scale. Addressing this implementation gap is as important as developing new interventions.

The evidence also questions any assumption that challenges are resolved once the early recovery period is complete. A 2025 systematic review and meta-analysis of 92 studies involving over 27,000 patients confirmed that those with an ostomy reported significantly more body image concerns and sexual dysfunction compared with those without, establishing these as enduring population-level sequelae rather than transient postoperative difficulties (113). Long-term survivors continue to face clothing and dietary restrictions, equipment management difficulties, and psychological challenges, with specialist nursing and patient association support remaining important throughout the full duration of survivorship (29, 34, 36, 46). Care models should therefore extend well beyond early post-surgical support to address the complete trajectory of adaptation, integrating psychosocial, sexual health, and self-management components across the long term.

Real lived experience research adds a dimension to this picture that clinical outcome data alone cannot capture. Beyond measurable complications, patients carry a continuous attentional burden: monitoring stoma output, planning bathroom access, adjusting clothing, and anticipating leakage or odor, all of which can disrupt daily life and social participation even when acute complications are infrequent (12, 27, 91, 114). Unpredictable output patterns and peristomal skin irritation further erode confidence and reduce engagement in everyday activities (12, 33, 87). Digital monitoring tools and leakage sensors can support early detection and may reduce anxiety about undetected leakage, but they also risk increasing cognitive load if poorly designed or inadequately supported (16, 41, 114, 115). Evidence from broader remote patient monitoring research confirms that limited device usability can produce inaccurate data when patients complete assessments without clinical supervision, potentially increasing rather than reducing burden in vulnerable groups (115). Integrating real lived experience perspectives into care pathway design is therefore essential to ensure that interventions enhance experiential stability and everyday confidence rather than simply adding clinical monitoring obligations (7, 114). The finding that ostomy care remains unevenly supported across patient groups further reinforces the need for continuous, longitudinal pathways rather than episodic models of care (84, 85, 116).

The economic evidence presented in this review points to a care system in which complications, not prevention, remain the primary driver of cost. Peristomal skin complications affect between 36% and 73% of patients and are directly associated with increased readmission rates, longer hospital stays, and escalating resource use, yet current pathways are not consistently interrupting this cycle despite a clear evidence base for preventive approaches (33). Preventive skin barrier interventions have been shown to reduce peristomal skin complications by 77% compared with standard care, with average cost savings of $140,000 USD per patient, though cost effectiveness varies significantly across health systems, moderated by a country’s gross domestic product and healthcare expenditure per capita (117). This context dependence is important: economic benefits of preventive strategies are not universal, and pathway design must account for local health system capacity and reimbursement structures. Nonetheless, the case for upstream investment is compelling, and multidisciplinary approaches with patient empowerment at their center represent the priority direction within value-based care frameworks (33).

Across several high-income countries, ostomy care faces growing pressure from cost-driven procurement that conflicts with patient-centered evidence. In the UK, reviews under the Innovative Devices and Access Pathway have raised concerns about standardization overriding clinical need (118). Similarly, Spain’s Royal Decree 90/2026 promotes efficiency but restricts access through eligibility criteria, lowest-price rules, and administrative control (119). In the US, Medicare’s Competitive Bidding Program has been criticized for prioritizing cost over appropriateness, reducing access, and worsening outcomes (120). Together, these trends highlight a disconnect between system-level cost containment and the need for individualized product selection, with evidence linking such misalignment to poorer clinical outcomes, increased healthcare use, and reduced self-management capacity (28, 121).

The environmental dimension of ostomy care represents an emerging but increasingly urgent area of concern. A mixed-methods study of certified wound and ostomy nurses found that the majority anticipated adverse effects of climate change on skin integrity and stomal complications and identified an urgent need for greater integration of environmental awareness into nursing practice (122). Life cycle assessment frameworks applied to analogous chronic disease pathways demonstrate that ongoing treatment demands – including device use, consumables, and follow-up services – generate cumulative and measurable carbon footprints across patient populations, reinforcing the case for pathway-level sustainability planning rather than product-level interventions alone (123). Shifting services closer to patients through home care and community-based settings reduces hospital dependence, lowers emissions, decreases procedural waste, and reduces reliance on single-use products (94, 111, 124, 125). Tensions may nonetheless arise between sustainability goals and patient choice, particularly regarding single-use versus reusable products, and these must be navigated carefully within pathway design (122, 125, 126).

### Expert opinion

The perspectives in this section represent the authors’ own clinical and health-systems judgment, informed by the evidence reviewed above, rather than a formal expert consensus process. While the literature provides valuable insights into ostomy outcomes, complications, and self-management, real-world implementation often depends on nuanced clinical judgment and systems-level coordination. This expert opinion is intended to contextualize the preceding findings and to complement, rather than substitute for, that empirical evidence by highlighting practical strategies, identifying gaps in current care pathways, and emphasizing longitudinal, patient-centered approaches that may help optimize outcomes and reduce patient burden over time.

Much of current practice still treats the ostomy as the primary object of care, with the person managing it treated as secondary. Peristomal skin health is a useful illustration: it is one of the most common and most preventable sources of complication in this review, yet it is still frequently addressed reactively rather than through proactive education. Shifting that emphasis, building peristomal health literacy into routine care rather than treating it as damage control, is illustrative of what person-first pathway design looks like in practice.

Operative technique matters, but it represents only one part of a more complex picture. Proper stoma siting and construction are essential for effective pouching and ease of care, yet outcomes are equally shaped by education, follow-up, psychosocial support, and access to expertise. These elements do not operate in isolation, and it is the responsibility of the entire healthcare team to ensure they are consistently delivered and integrated across the care pathway.

Education is too often concentrated in the preoperative and immediate postoperative periods, precisely when patients are overwhelmed and least able to absorb new information. Teaching may be completed, but learning is frequently incomplete. A more effective model treats education as a longitudinal process in which core concepts introduced early are reinforced over time, with follow-up visits building skills, confidence, and independence rather than simply assessing outcomes. Caregivers and loved ones should be deliberately included in this process, as they often carry a central role in day-to-day management, particularly in the early postoperative period or in more vulnerable patients.

Reducing patient burden should be a central design principle of ostomy care pathways. Patients are simultaneously required to master new technical skills, navigate complex supply systems, and adapt to the psychosocial impact of a changed body – a convergence of demands that can overwhelm even well-supported individuals. Cognitive burden can be reduced through simplified education and a focus on pattern recognition rather than isolated procedural tasks. Logistical burden is best addressed through standardized discharge pathways and reliable early follow-up. Emotional burden benefits from normalization, peer support, and the routine integration of behavioral health and advocacy resources into care, rather than treating these as optional additions.

Self-management should be understood as more than technical proficiency. Beyond changing an appliance, patients must learn to anticipate problems, recognize early signs of complications, and respond appropriately. Scenario-based teaching can prepare patients for common challenges such as peristomal skin irritation, leakage, or changes in output. These competencies develop over time and require reinforcement well beyond the hospital setting, which is why access to specialist support must extend into the community. As summarized in the role of specialist ostomy nursing section above, wound, ostomy, and continence nurses play a central role in optimizing pouching, troubleshooting complications, and supporting patient adaptation, yet access to this expertise remains inconsistent across health systems. Expanding the ostomy nursing workforce and ensuring timely patient access should be a priority for any system serious about improving outcomes.

Most ostomy-related complications are predictable and, in many cases, preventable. Peristomal skin complications, dehydration in ileostomy patients, and appliance-related failures account for a disproportionate share of morbidity and healthcare utilization. Early post-discharge contact, low-threshold access to specialist support, and systematic identification of high-risk patients can shift care from reactive to proactive, reducing emergency presentations and readmissions that too often reflect gaps in outpatient support rather than inevitable disease progression.

Attention to resource use is also warranted. Ostomy care is inherently supply intensive, but unnecessary waste is frequently driven by poor appliance fit, inadequate education, and preventable complications. Improvements in pouching reliability and complication prevention enhance both patient experience and material efficiency, creating a pathway to more sustainable care delivery.

This efficiency argument connects to a broader systemic concern. Psychological morbidity among ostomy patients places incremental demand on mental health services that are already under significant strain, and the nursing workforce that supports these patients is itself operating within a system facing serious capacity pressures. World Health Organization (WHO) data confirm that at least a quarter of health and care workers globally reported symptoms of anxiety, depression, and burnout between 2020 and 2022 (57), with no meaningful improvement since, reflecting a workforce in crisis (56).

We contend that developing robust ostomy self-management pathways is not simply a quality-of-care issue but a workforce strategy. The WHO projects a global shortfall of 10 million health workers by 2030 (56), with nurses disproportionately represented in that deficit due to escalating workloads, burnout, and attrition that current systems are failing to address (127). Care models that genuinely build patient self-efficacy, reducing reactive and unplanned contact and enabling nurses to focus on higher-complexity clinical needs, are not a peripheral efficiency gain. They are a meaningful contribution to workforce sustainability, staff well-being, and the long-term quality of care that health systems can deliver.

Looking forward, care delivery should align with established patient-centered standards, including the UOAA Patient Bill of Rights (24). These frameworks define high-quality ostomy care and provide structure for implementation across settings while creating a foundation for integrating emerging tools, including artificial intelligence-supported education and triage systems. Such approaches can extend access to reliable, real-time guidance while maintaining consistency with best practices. At the same time, research infrastructure must expand. Large, collaborative registries would allow rigorous evaluation of operative techniques and their downstream impact on QoL, complications, and healthcare utilization.

In our view, future advances in ostomy care are unlikely to come from devices alone; they will arise chiefly from designing systems that support patients longitudinally, anticipate common challenges, and make high-quality care easier to deliver and to receive.

### Limitations

This review has several limitations. It is a narrative rather than a systematic review: study selection and synthesis, while guided by pre-specified eligibility criteria and thematic domains, were not conducted using dual independent screening, a PRISMA flow diagram, or formal risk-of-bias or GRADE appraisal, and findings should be interpreted as a qualitative synthesis of the available literature rather than a quantified, quality-weighted estimate of effect. The included evidence is heterogeneous in design, population, and outcome measurement, spans multiple healthcare systems with differing care models and reimbursement structures, and is predominantly observational; few findings are supported by randomized controlled trials or meta-analysis. Finally, the environmental sustainability evidence is particularly sparse and largely extrapolated from non-ostomy-specific healthcare data and should be considered hypothesis-generating rather than conclusive.

## Conclusion

The long-term burden of ostomy self-management is substantial, persistent, and multidimensional. Complications are common, adaptation is non-linear, and the gap between evidence and real-world implementation remains wide. The evidence reviewed suggests that episodic, reactive care is often insufficient, and that longitudinal, patient-centered pathways integrating structured education, specialist nursing, psychosocial support, and digital tools offer a more promising approach across the full arc of the patient journey. When coherently aligned, these elements have been associated with fewer complications and readmissions and with lower costs and environmental impact, though the strength of this evidence varies by domain and further prospective research is needed, particularly on environmental outcomes.

The evidence reviewed here supports a simple reframing of priorities: care pathways should be designed to help people live well with an ostomy, not simply to manage the ostomy itself and its downstream consequences. Achieving this requires action at multiple levels: clinicians moving toward person-centered models that account for lived experience; health systems investing in specialist access and pathway infrastructure; and industry engaging seriously with product sustainability and innovation. Future research should prioritize longitudinal, real-world studies capturing clinical and experiential outcomes, with particular attention to underserved populations and the long-term economic and environmental case for preventive care. Embedding ostomy management within a broader chronic care ecosystem – one that integrates digital innovation, psychosocial support, and equitable access – offers a model that is more effective for patients and more sustainable for the systems that serve them.

## References

[1] Hidden Disabilities Sunflower . Ostomy (2026). Available online at: https://hdsunflower.com/row/insights/post/ostomy (Accessed February 4, 2026).

[2] World Cancer Research Fund . Colorectal Cancer Statistics (2026). Available online at: https://www.wcrf.org/preventing-cancer/cancer-statistics/colorectal-cancer-statistics/ (Accessed February 4, 2026).

[3] Tsujinaka S Kawana K Okamoto K Aizawa H Mizusawa J . Current management of intestinal stomas and their complications. J Anus Rectum Colon. (2020) 4:25–33. doi: 10.23922/jarc.2019-032 32002473 PMC6989127

[4] Solitano V Vuyyuru SK Yuan Y Jairath V . Management of complications in patients with an ileostomy: an umbrella review of systematic reviews for the EndOTrial Consortium. Int J Colorectal Dis. (2024) 39:147. doi: 10.1007/s00384-024-04714-8 39304546 PMC11415412

[5] Kang Y Li X Zhang Y Wang Y . Life experience of patients living with urostomy: a meta-synthesis of qualitative research. Psychooncology. (2025) 34:e70096. doi: 10.1002/pon.70096 40080434

[6] Alenezi A McGrath I Kimpton A Livesay K . Quality of life among ostomy patients: a narrative literature review. J Clin Nurs. (2021) 30:3111–23. doi: 10.1111/jocn.15840 33982291

[7] Heydari A Manzari ZS Pouresmail Z . Nursing intervention for quality of life in patients with ostomy: a systematic review. Iran J Nurs Midwifery Res. (2023) 28:371–83. doi: 10.4103/ijnmr.ijnmr_266_22 37694203 PMC10484385

[8] Wang SM Zhang L Liu Y Chen X Li J Zhou Y . Qualitative exploration of home life experiences and care needs among elderly patients with temporary intestinal stomas. World J Gastroenterol. (2024) 30:2893–901. doi: 10.3748/wjg.v30.i22.2893 38947295 PMC11212711

[9] Ercolano E Safar B Doyle E McCorkle R Knobf MT Lazenby M . Self-management goals of cancer survivors with an ostomy. J Cancer Surviv. (2023) 17:1480–7. doi: 10.1007/s11764-022-01164-5 35522352

[10] Toma E Ramage C Villa G Skountrianos G Nichols T . A holistic view of the stoma care pathway in Italy: a nationwide learning survey. Gastrointest Nurs. (2022) 20:S24–33. doi: 10.12968/gasn.2022.20.sup4.s24

[11] van Pelt K Lambrecht L Sergeant G Wolthuis A . Effects of a perioperative educational pathway on ostomy self-care, level of independence and need for visiting nurse services: a comparative observational cohort study. Colorectal Dis. (2024) 26:1258–65. doi: 10.1111/codi.17044 38807266

[12] Haughey M Neyens DM Hopkins CS Gonzaga C Harman M . Identifying strategies for home management of ostomy care: content analysis of YouTube. JMIR Hum Factors. (2025) 12:e66634. doi: 10.2196/66634 40053741 PMC11926463

[13] Ma LL Zhang YJ Yao JX Zhang WY Zhuang HR . Influencing factors associated with peristomal skin complications after colorectal ostomy surgery: a systematic review and meta-analysis. Wound Manag Prev. (2024) 70. doi: 10.25270/wmp.23114 39361350

[14] Qiao J Zhao Y Lu Y Li Q Dong HJ . Assessing the impact of educational eHealth and mHealth interventions on health outcomes in continuity of care for enterostomy patients: a meta-analysis. Eur J Oncol Nurs. (2024) 72:102676. doi: 10.1016/j.ejon.2024.102676 39241275

[15] Goodman W Buswell M Griffiths J Radley S . A systematic review and meta-analysis of the effectiveness of self-management interventions in people with a stoma. J Adv Nurs. (2022) 78:722–38. doi: 10.1111/jan.15085 34708416

[16] Brady RRW Hawkins N Foo I Sherwood A Paterson H Boyd K . Evaluating the effect of a novel digital ostomy device on leakage incidents, quality of life, mental well-being, and patient self-care: an interventional, multicentre clinical trial. J Clin Med. (2024) 13:5673. doi: 10.3390/jcm13195673 39407731 PMC11477101

[17] Danielsen A . Life after stoma creation. Dan Med J. (2013) 60:B4732. 24083536

[18] Sun V Grant M Wendel CS McMullen CK Bulkley JE Herrinton LJ . Ostomy telehealth for cancer survivors: design of the Ostomy Self-management Training (OSMT) randomized trial. Contemp Clin Trials. (2018) 64:167–72. doi: 10.1016/j.cct.2017.10.008 29051047 PMC6252251

[19] Licciardello A Bonetti L Cappellini C . Experiences and perceptions of people living with a colostomy for a long time: a phenomenological study. Recenti Prog Med. (2020) 111:149–53. doi: 10.1701/3315.32856 32157262

[20] The Asia and South Pacific Ostomy Association . About Aspoa (2026). Available online at: https://ostomyasiasouthpacific.org/ (Accessed February 4, 2026).

[21] European Ostomy Association . Mission, Vision, & Values (2026). Available online at: https://ostomyeurope.org/ (Accessed February 4, 2026).

[22] International Ostomy Association . The IOA Visitors Programme (2026). Available online at: http://ostomyinternational.org/ (Accessed February 4, 2026).

[23] The Ostomy Association of the Americas . About Us (2026). Available online at: https://www.ostomyamericas.org/aboutoaa (Accessed February 4, 2026).

[24] United Ostomy Associations of America . Support, Education, Resources, Advocacy (2026). Available online at: https://www.ostomy.org/ (Accessed February 4, 2026).

[25] McNichol L Markiewicz A Goldstine J Nichols TR . A cross-sectional survey reporting on the value of patient-centered ostomy programs: a smooth transition after ostomy surgery. J Wound Ostomy Continence Nurs. (2022) 49:449–54. doi: 10.1097/WON.0000000000000907 36108228 PMC9481286

[26] Nizum N Jacob G . Systematic review of ostomy care pathways. Adv Skin Wound Care. (2022) 35:290–5. doi: 10.1097/01.ASW.0000823976.96962.b6 35442921

[27] Bulkley JE McMullen CK Grant M Wendel C Hornbrook MC Krouse RS . Ongoing ostomy self-care challenges of long-term rectal cancer survivors. Support Care Cancer. (2018) 26:3933–9. doi: 10.1007/s00520-018-4268-0 29845420 PMC6160331

[28] Carlsson E Forsmark A Sternhufvud C Scheffel G Andersen FB Persson EI . Short- and long-term direct and indirect costs of illness after ostomy creation: a Swedish nationwide registry study. BMC Health Serv Res. (2023) 23:837. doi: 10.1186/s12913-023-09850-5 37553576 PMC10408161

[29] Grant M McMullen CK Altschuler A Mohler MJ Hornbrook MC Herrinton LJ . Cancer survivors' greatest challenges of living with an ostomy: findings from the Ostomy Self-Management Telehealth (OSMT) randomized trial. Support Care Cancer. (2022) 30:1139–47. doi: 10.1007/s00520-021-06449-6 34435212

[30] Martin-Gil B Sanchez-Gomez C Martin-Rodero H Casado-Espejo F Rodriguez-Martin B . Changes in the quality of life of adults with an ostomy during the first year after surgery as part of the Best Practice Spotlight Organisation Programme. Int Wound J. (2024) 21:e14456. doi: 10.1111/iwj.14456 37963817 PMC10898385

[31] Krogsgaard M Thomsen T Vinther A Lauridsen SV Ellebaek MB Gogenur I . Life with a stoma across five European countries: a cross-sectional study on long-term rectal cancer survivors. Support Care Cancer. (2022) 30:8969–79. doi: 10.1007/s00520-022-07293-y 35930059

[32] Jayarajah U Samarasekera DN . A cross-sectional study of quality of life in a cohort of enteral ostomy patients presenting to a tertiary care hospital in a developing country in South Asia. BMC Res Notes. (2017) 10:75. doi: 10.1186/s13104-017-2406-2 28137299 PMC5282704

[33] D'Ambrosio F Pappalardo C Scardigno A Maida A Ricciardi R Calabro GE . Peristomal skin complications in ileostomy and colostomy patients: what we need to know from a public health perspective. Int J Environ Res Public Health. (2023) 20:79. doi: 10.3390/ijerph20010079 36612395 PMC9819694

[34] Al Habash K Turfa R Jaddoua S Al Rabayah A . Measuring health-related quality of life of patients with metastatic colorectal cancer using the Jordanian EQ-5D-3L value set: a cross-sectional observational study. BMJ Open. (2025) 15:e099635. doi: 10.1136/bmjopen-2025-099635 41248350 PMC12588013

[35] Fingren J Lindholm E Petersen C Hallen A-M Carlsson E . A prospective, explorative study to assess adjustment 1 year after ostomy surgery among Swedish patients. Ostomy Wound Manage. (2018) 64:12–22. doi: 10.25270/owm.2018.6.1222 30059344

[36] Sun V Grant M McMullen CK Altschuler A Mohler MJ Hornbrook MC . Surviving colorectal cancer. J Wound Ostomy Continence Nurs. (2013) 40:61–72. doi: 10.1097/WON.0b013e3182750143 23222968 PMC3536890

[37] Semi H Sambari SS Syam Y Irwan AM . Effect of follow-up telephone by enterostomal nurses on patients with permanent colostomy: a systematic review. Bangladesh J Med Sci. (2022) 21:54–66. doi: 10.3329/bjms.v21i1.56327 40208441

[38] Murken DR Bleier JIS . Ostomy-related complications. Clin Colon Rectal Surg. (2019) 32:176–82. doi: 10.1055/s-0038-1676995 31061647 PMC6494607

[39] Babakhanlou R Larkin K Hita AG Stroh J Yeung S-C . Stoma-related complications and emergencies. Int J Emerg Med. (2022) 15:17. doi: 10.1186/s12245-022-00421-9 35534817 PMC9082897

[40] Malik T Lee M Harikrishnan A . The incidence of stoma related morbidity: a systematic review of randomised controlled trials. Ann R Coll Surg Engl. (2018) 100:501–8. doi: 10.1308/rcsann.2018.0126 30112948 PMC6214073

[41] Brady RR Foo I Hawkins N Sherwood A Paterson H Boyd K . Novel digital leakage notification system can promote better stoma management routines: a multicentre clinical trial. Br J Nurs. (2025) 34:14–22. doi: 10.12968/bjon.2025.0278 39792106

[42] Grant M McMullen CK Altschuler A Mohler MJ Hornbrook MC Herrinton LJ . Gender differences in quality of life among long-term colorectal cancer survivors with ostomies. Oncol Nurs Forum. (2011) 38:587–96. doi: 10.1188/11.ONF.587-596 21875846 PMC3251903

[43] Nehemiah A Sun V Krouse RS Grant M Hornbrook MC McMullen CK . Intimacy and survivors of cancer with ostomies: findings from the Ostomy Self-management Training trial. J Sex Med. (2023) 20:1319–24. doi: 10.1093/jsxmed/qdad119 37769350 PMC10627782

[44] Indrebo KL Aasprang A Olsen TE Andersen JR . Experiences and results from using a novel clinical feedback system in routine stoma care nurse follow-up of patients with an ostomy: a longitudinal study. J Patient Rep Outcomes. (2023) 7:27. doi: 10.1186/s41687-023-00573-z 36913078 PMC10010226

[45] Harris MS Kelly K Parise C . Does preoperative ostomy education decrease anxiety in the new ostomy patient? A quantitative comparison cohort study. J Wound Ostomy Continence Nurs. (2020) 47:137–9. doi: 10.1097/WON.0000000000000623 32150139

[46] Sun V Bojorquez O Raza S Grant M Hornbrook MC Krouse RS . Cancer survivors' challenges with ostomy appliances and self-management. J Clin Oncol. (2019) 37:e23091. doi: 10.1200/JCO.2019.37.15_suppl.e23091 42148471

[47] Antequera-Antequera A Rueda-Medina B Garcia-Lopez MV Fernandez-Sola C Hernandez-Padilla JM Lopez-Rodriguez MM . Home-based, telematic gradual exercise for permanent colostomy patients: protocol for a randomized controlled trial. Healthcare. (2025) 13:2742. doi: 10.3390/healthcare13212742 41228112 PMC12607481

[48] Kim YM Jang HJ Lee YJ . The effectiveness of preoperative stoma site marking on patient outcomes: a systematic review and meta-analysis. J Adv Nurs. (2021) 77:4332–46. doi: 10.1111/jan.14915 34118170

[49] Khalilzadeh Ganjalikhani M Tirgari B Roudi Rashtabadi O Shahesmaeili A . Studying the effect of structured ostomy care training on quality of life and anxiety of patients with permanent ostomy. Int Wound J. (2019) 16:1383–90. doi: 10.1111/iwj.13201 31419023 PMC7948913

[50] Hao J Xu Y Li H . The value of applying a continuous nursing model based on virtual platforms for patients with colostomy or ileostomy. Adv Skin Wound Care. (2023) 36:206–12. doi: 10.1097/01.ASW.0000919960.94295.53 36940377 PMC10026954

[51] Burge KG Sheffer HF Smithson M McLeod C Chu D Hollis RH . Expedited discharge and risk of readmission after ostomy construction. Surgery. (2025) 178:108948. doi: 10.1016/j.surg.2024.10.031 39617648 PMC11717625

[52] Eid MA Curcio A Lacroix E Caiazzo R Zarzaur BL Ogilvie JW . Reducing new ileostomy readmissions in a rural health care setting: a quality improvement initiative. Dis Colon Rectum. (2022) 65:928–35. doi: 10.1097/DCR.0000000000002142 34775414

[53] Rojanasarot S . The impact of early involvement in a postdischarge support program for ostomy surgery patients on preventable healthcare utilization. J Wound Ostomy Continence Nurs. (2017) 44:160–7. doi: 10.1097/WON.0000000000000395 29189646 PMC5757661

[54] Lin L Fang Y Wei Y Huang F Zheng J Xiao H . The effects of a nurse-led discharge planning on the health outcomes of colorectal cancer patients with stomas: a randomized controlled trial. Int J Nurs Stud. (2024) 155:104769. doi: 10.1016/j.ijnurstu.2024.104769 38676992

[55] Rock MC Sun V Krouse RS Grant M Hornbrook MC McMullen CK . Adapting to the burdens of care: a telehealth program for cancer survivors with ostomies. Support Care Cancer. (2023) 31:15. doi: 10.1007/s00520-022-07461-0 36513895

[56] World Health Organization . Mental Health Atlas 2024 (2025). Available online at: https://www.who.int/publications/i/item/9789240114487 (Accessed March 31, 2026).

[57] World Health Organization . Protecting Health and Care Workers' Mental Health and Well-Being: Technical Consultation Meeting (2024). Available online at: https://www.who.int/news/item/25-04-2024-202404_protecthw_mentalhealth (Accessed March 31, 2026).

[58] van Loon YT Clermonts S Belt R Nagle D Wasowicz DK Zimmerman DDE . Implementation of an easy in-hospital educational stoma pathway results in decrease of home nursing care services after discharge. Colorectal Dis. (2020) 22:1175–83. doi: 10.1111/codi.15034 32180331

[59] Davis BR Ariyan CE Berger DL Bordeianou LG Chang GJ Feingold DL . The American Society of Colon and Rectal Surgeons clinical practice guidelines for ostomy surgery. Dis Colon Rectum. (2022) 65:1173–90. doi: 10.1097/DCR.0000000000002498 35616386

[60] Forsmo HM Pfeffer F Rasdal A Sintonen H Korner H Erichsen C . Pre- and postoperative stoma education and guidance within an enhanced recovery after surgery (ERAS) programme reduces length of hospital stay in colorectal surgery. Int J Surg. (2016) 36:121–6. doi: 10.1016/j.ijsu.2016.10.031 27780772

[61] Song Q Yin G Guo X Lv X Yu K Liu C . Effects of a self-management program for patients with colorectal cancer and a colostomy. J Wound Ostomy Continence Nurs. (2021) 48:311–7. doi: 10.1097/WON.0000000000000779 34186549

[62] Xu L Zhou MZ . Effect of internet multiple linkage mode-based extended care combined with in-hospital comfort care on colorectal cancer patients undergoing colostomy. World J Gastrointest Surg. (2023) 15:1959–68. doi: 10.4240/wjgs.v15.i9.1959 37901742 PMC10600758

[63] Chen HL Tu MH . The effectiveness of development mHealth apps on the self-care ability of patients with ostomy. Stud Health Technol Inform. (2024) 315:569–70. doi: 10.3233/SHTI240220 39049326

[64] Soares-Pinto I Braga AMP Santos I Ferreira N Silva S Alves PJ . eHealth promoting stoma self-care for people with an elimination ostomy: focus group study. JMIR Hum Factors. (2023) 10:e39826. doi: 10.2196/39826 36912879 PMC10132022

[65] Ko HF Wu MF Lu JZ . A randomized control study: the effectiveness of multimedia education on self-care and quality of life in patients with enterostomy. Int Wound J. (2023) 20:4244–52. doi: 10.1111/iwj.14326 37488713 PMC10681488

[66] Kim S Jeong HN . Examining the impact of telehealth stoma care interventions on the ostomates: a systematic review and meta-analysis. J Clin Nurs. (2025) 35:621–630. doi: 10.1111/jocn.70037. advance online publication. 40626636 PMC12779166

[67] Al Daken LI Ahmad MM . The implementation of mindfulness-based interventions and educational interventions to support family caregivers of patients with cancer: a systematic review. Perspect Psychiatr Care. (2018) 54:441–52. doi: 10.1111/ppc.12286 29745417

[68] Lim SH Chan SWC Chow A Zhu L Lai JH He HG . Pilot trial of a STOMA psychosocial intervention programme for colorectal cancer patients with stomas. J Adv Nurs. (2019) 75:1338–46. doi: 10.1111/jan.13973 30740765

[69] Faury S . Implementation of psychosocial intervention StomieCare for patients with rectal cancer treated by rectal excision and temporary stoma: a pilot study of feasibility, acceptability and efficacy. Psychooncologie. (2023) 17:147–57. doi: 10.32604/po.2023.044901

[70] Martin-Munoz B Montesinos-Galvez AC Crespillo-Diaz AY Jodar-Sanchez F . Efficacy of a social interaction intervention in early postoperative period to improve coping in persons with an ostomy: a randomized controlled trial. J Wound Ostomy Continence Nurs. (2022) 49:352–7. doi: 10.1097/WON.0000000000000886 35809011

[71] Sun V Grant M McMullen CK Altschuler A Mohler MJ Hornbrook MC . From diagnosis through survivorship: health-care experiences of colorectal cancer survivors with ostomies. Support Care Cancer. (2014) 22:1563–70. doi: 10.1007/s00520-014-2118-2 24442998 PMC4130212

[72] Zhang X Zhou L Zhang F Li H Wang L Wang Z . Network analysis of psychosocial adaptation in intestinal stoma patients: a national cross-sectional study in China. J Adv Nurs. (2026) 82:1243–52. doi: 10.1111/jan.16928 40135956

[73] Koc MA Akyol C Gokmen D Aydin D Erkek AB Kuzu MA . Effect of prehabilitation on stoma self-care, anxiety, depression, and quality of life in patients with stomas: a randomized controlled trial. Dis Colon Rectum. (2023) 66:138–47. doi: 10.1097/DCR.0000000000002275 35195553

[74] Staruchowicz L Taylor A . Effectiveness of follow-up care provided by stoma care nurses: a systematic review protocol. JBI Database Syst Rev Implement Rep. (2012) 10:1–9. doi: 10.11124/jbisrir-2012-1600 27820457

[75] Xue Y Lv K Yuan C Fan G Yu P . The experience and needs of self-care in elderly colorectal cancer stoma patients: a qualitative study. Support Care Cancer. (2025) 33:474. doi: 10.1007/s00520-025-09530-6 40372572

[76] Lopez E Pittman J Rawl S . (2016). “ Complications and stoma care self-efficacy are associated with ostomy adjustment in people with an intestinal or urinary ostomy”, in: Conference presentation. Available online at: https://scholarworks.indianapolis.iu.edu/items/8ee77045-5f80-4ce9-91cb-e64c82538ee8 (Accessed January 7, 2026).

[77] Zelga P Kluska P Zelga M Piasecka-Zelga J Dziki A . Patient-related factors associated with stoma and peristomal complications following fecal ostomy surgery: a scoping review. J Wound Ostomy Continence Nurs. (2021) 48:415–30. doi: 10.1097/WON.0000000000000796 34495932

[78] Sheffer HF Burge KG Smithson M McLeod C Chu D Hollis RH . High risk populations for unplanned healthcare utilization following ostomy construction. Am J Surg. (2025) 239:115799. doi: 10.1016/j.amjsurg.2024.115799 38890038 PMC11638406

[79] Marcomini I Iovino P Rasero L Manara DF Vellone E Villa G . Self-care and quality of life of ostomy patients: a structural equation modeling analysis. Nurs Rep. (2024) 14:3417–26. doi: 10.3390/nursrep14040247 39585138 PMC11587398

[80] Liu C Song Q Qu Y Yin G Wang J Lv X . Course and predictors of supportive care needs among colorectal cancer survivors with ostomies: a longitudinal study. Support Care Cancer. (2024) 32:395. doi: 10.1007/s00520-024-08607-y. advance online publication. 38816568

[81] Liu F Diao W Huang H Guo W Luo X . Factors influencing quality of life in patients with a colostomy due to rectal cancer: a retrospective cohort study with multivariate and subgroup analyses. Oncol Lett. (2025) 29:295. doi: 10.3892/ol.2025.15041 40271003 PMC12016008

[82] Giordano V Nicolotti M Corvese F Vellone E Alvaro R Villa G . Describing self-care and its associated variables in ostomy patients. J Adv Nurs. (2020) 76:2982–92. doi: 10.1111/jan.14499 32844474

[83] Brady RRW Hawkins N Foo I Sherwood A Paterson H Boyd K . The prevalence of leakage, peristomal skin complications and impact on quality of life in the first year following stoma surgery. Nurs Rep. (2025) 15:107. doi: 10.3390/nursrep15030107 40137682 PMC11946121

[84] Mo J Wendel CS Bulkley JE McMullen CK Grant M Hornbrook MC . Healthy behaviors are associated with positive outcomes for cancer survivors with ostomies: a cross-sectional study. J Cancer Surviv. (2021) 15:461–9. doi: 10.1007/s11764-020-00940-5 32940890 PMC7965775

[85] Li L Liu L Kang H Zhang L . The influence of predictive nursing on the emotions and self-management abilities of post-colostomy rectal cancer patients. Am J Transl Res. (2021) 13:6543–51. PMC8290706 34306395

[86] Duque PA Valencia Rico CL Campino Valderrama SM Lopez Gonzalez LA . Effects of socio-educational interventions on the quality of life of people with a digestive ostomy. SAGE Open Nurs. (2023) 9:23779608231177542. doi: 10.1177/23779608231177542 37255580 PMC10226308

[87] Simpson E Sheridan E Thompson J Rance J Bhatt N Durai D . Living with and without an intestinal stoma: factors that promote psychological well-being and self-care: a cross-sectional study. Nurs Open. (2023) 10:7811–25. doi: 10.1002/nop2.2030 37840444 PMC10643826

[88] Zuluaga Sarmiento LM Carrillo Gonzalez GM . Self-management experience in adults with colostomies due to colorectal cancer. Rev Cuidarte. (2025) 16:e4265. doi: 10.15649/cuidarte.4265 40927162 PMC12400984

[89] Wen S Ye X Guo P Lu Q Ding H Tian J . Effects of transtheoretical model-based intervention on the self-management of patients with an ostomy: a randomised controlled trial. J Clin Nurs. (2019) 28:4312–21. doi: 10.1111/jocn.14731 30549366

[90] Asefa T Shum E Siahpush M Smith D Belete H . The impact of ostomy on colorectal cancer patients and caregivers: a qualitative study. Support Care Cancer. (2025) 33:1030. doi: 10.1007/s00520-025-10075-x 41204028 PMC12594726

[91] Soelling SJ Bednarski BK Messick CA You YN Skibber JM Rodriguez-Bigas M . Challenges faced by patients undergoing fecal ostomy surgery: a qualitative study of colorectal cancer patient perspectives. J Gastrointest Surg. (2025) 29:101963. doi: 10.1016/j.gassur.2025.101963 39824243 PMC13134573

[92] Stavropoulou A Rovithis M Kelesi M Vasilopoulos G Sigala E Papageorgiou D . Living with a stoma: exploring the lived experience of patients with permanent colostomy. Int J Environ Res Public Health. (2021) 18:8512. doi: 10.3390/ijerph18168512 34444262 PMC8393572

[93] Morkhagen AE Nortvedt L . A qualitative study on how younger women experience living with an ostomy. Int J Environ Res Public Health. (2023) 20:5627. doi: 10.3390/ijerph20095627 37174147 PMC10178502

[94] Andersen FB Kjellberg J Ibsen R Sternhufvud C Petersen B . The clinical and economic burden of illness in the first two years after ostomy creation: a nationwide Danish cohort study. Expert Rev Pharmacoecon Outcomes Res. (2024) 24:567–75. doi: 10.1080/14737167.2024.2324047 38433657

[95] Maeng D Hoffman RL Sun V Sticca RP Krouse RS . Post-surgical acute care utilization and cost of care among cancer survivors with an ostomy: findings from three large hospital systems in the United States. J Cancer Policy. (2025) 43:100534. doi: 10.1016/j.jcpo.2024.100534 39657389 PMC11890926

[96] Brady RRW Foo I Hawkins N Sherwood A Paterson H Boyd K . Complications and healthcare costs associated with the first year following colostomy and ileostomy formation: a retrospective study. J Wound Ostomy Continence Nurs. (2023) 50:475–83. doi: 10.1097/WON.0000000000001028 37966075

[97] Mthombeni F Cawson M Chan G Boisen EB Rethmeier LO Pearson-Stuttard J . The economic burden of stomas in the UK: a retrospective observational study of health records and hospital encounters. Br J Nurs. (2023) 32:S12–20. doi: 10.12968/bjon.2023.32.22.S12 38060389

[98] Rolls N Creedon S Draper S Johnston C Kelly G Kilpatrick M . Healthcare resource use and associated costs for patients with an ileostomy experiencing peristomal skin complications. Int Wound J. (2023) 20:2540–50. doi: 10.1111/iwj.14118 37020423 PMC10410323

[99] Hollister Incorporated . Skin First: Counting the Cost of Peristomal Skin Complications (2024). Available online at: https://www.hollister.co.uk/-/media/files/pdfs-for-download/uk/frontier-costs-of-peristomal-skin-complications-launch-02-10-24.ashx (Accessed March 19, 2026).

[100] HMP Global Learning Network . Ostomy Supply Heroes: How Financial Distress After Ostomy Surgery Inspired Four Ostomates to Create Low-Cost Ostomy Supply Programs (2020). Available online at: https://www.hmpgloballearningnetwork.com/site/wmp/article/ostomy-supply-heroes (Accessed March 30, 2026).

[101] Grand View Research . Ostomy Care and Accessories Market (2025-2030) (2026). Available online at: https://www.grandviewresearch.com/industry-analysis/stoma-care-ostomy-care-accessories-market (Accessed February 4, 2026).

[102] Montesinos Galvez AC Martin-Munoz B Jodar-Sanchez F Molina Linde JM Villegas Portero R Crespillo-Diaz AY . Value-based healthcare in ostomies. Int J Environ Res Public Health. (2020) 17:5879. doi: 10.3390/ijerph17165879 32823745 PMC7460258

[103] Jin Y Tian X Li Y Jimenez-Herrera M Wang H . Effects of continuous care on health outcomes in patients with stoma: a systematic review and meta-analysis. Asia Pac J Oncol Nurs. (2022) 9:21–31. doi: 10.1016/j.apjon.2021.12.006 35528792 PMC9072188

[104] Cidav Z Mandell DS Pyne JM Beidas RS Curran G Marcus SC . Programmatic costs of the Telehealth Ostomy Self-Management Training: an application of time-driven activity-based costing. Value Health. (2021) 24:1245–53. doi: 10.1016/j.jval.2021.03.018 34452703

[105] Gilpin V Williams J Connolly P Dunne N . Evolution of ostomy pouch design: opportunities for composite technologies to advance patient care. J Compos Sci. (2024) 8:388. doi: 10.3390/jcs8100388 30654563

[106] Cho Y Kim H Park J Lee S Choi J . Achieving the sustainable waste management of medical plastic packaging using a life cycle assessment approach. Heliyon. (2024) 10:e38185. doi: 10.1016/j.heliyon.2024.e38185 39398058 PMC11466672

[107] Watts N Amann M Arnell N Ayeb-Karlsson S Beagley J Belesova K . The 2020 report of the Lancet countdown on health and climate change: responding to converging crises. Lancet. (2021) 397:129–70. doi: 10.1016/S0140-6736(20)32290-X 33278353 PMC7616803

[108] Cristiano W De Marchi C Di Domenico K Punzo O Mancini A Mancini L . The elephant in the room in greenhouse gases emissions: rethinking healthcare systems to face climate change. Environ Sci Eur. (2024) 36. doi: 10.1186/s12302-024-00839-3 38164791

[109] Leal Filho W Luetz JM Thanekar UD Dinis MAP Forrester M . Climate-friendly healthcare: reducing the impacts of the healthcare sector on the world's climate. Sustain Sci. (2024) 19:1103–9. doi: 10.1007/s11625-024-01487-5 30311153

[110] Hu D Hahn M Dorfman D Martin KD Moretti K . The financial and environmental impact of unopened medical supplies discarded in the emergency department. Am J Emerg Med. (2024) 83:109–13. doi: 10.1016/j.ajem.2024.06.022 39002496

[111] Tyler N Wright N Waring J Farquhar M . Transitional care interventions from hospital to community to reduce health care use and improve patient outcomes. JAMA Netw Open. (2023) 6:e2344825. doi: 10.1001/jamanetworkopen.2023.44825 38032642 PMC10690480

[112] Sheffer HF Burge KG Hollis RH Porterfield JR Richman JS Chu DI . Defining opportunities to improve perioperative ostomy care and education. Ann Surg Open. (2025) 6:e563. doi: 10.1097/AS9.0000000000000563 40134481 PMC11932619

[113] Redeker C Grunfeld E Miles A . The impact of an ostomy on body image and sexual function of patients with colorectal cancer: a systematic review and meta-analysis. Psychooncology. (2025) 34:e70249. doi: 10.1002/pon.70249 40754638

[114] Umio Limited . Self-Management, Evidence and Life With a Stoma. Internal Report, Version 1.0. Oxford: Umio Limited (2026).

[115] Baumann S Stone RT Abdelall E . Introducing a remote patient monitoring usability impact model to overcome challenges. Sensors. (2024) 24:3977. doi: 10.3390/s24123977 38931760 PMC11207983

[116] Sayar S Vural F . Effects of a support group intervention on adaptation to ostomy, quality of life, and complication severity in patients with an ostomy. J Wound Ostomy Continence Nurs. (2025) 52:485–491. doi: 10.1097/WON.0000000000001118 41235849

[117] Caruso R Arrigoni C Magon A Conte G Canzan F Ferri P . Evaluating the clinical and economic impact of ceramide-infused skin barriers in patients with intestinal and urinary stomas: a systematic review and meta-analysis. Clin Ther. (2025) 47:e21–32. doi: 10.1016/j.clinthera.2025.02.001 40059006

[118] National Institute for Health and Care Excellence . One-Piece Closed Bags for Colostomies: Late-Stage Assessment. Health Technology Evaluation HTE29 (2025). Available online at: https://www.nice.org.uk/guidance/hte29 (Accessed April 27, 2026).

[119] Ministerio de Sanidad . The Government Approves a Royal Decree to Modernize the Financing of Health Products in Pharmaceutical Benefits (2026). Available online at: https://www.sanidad.gob.es/gabinete/notasPrensa.do?metodo=detalle&id=6848 (Accessed April 27, 2026).

[120] United Ostomy Associations of America . Understanding the Medicare Competitive Bidding Ruling (2026). Available online at: https://www.ostomy.org/understanding-the-medicare-competitive-bidding-proposal/ (Accessed April 27, 2026).

[121] Schaps D . Medicare is restricting care for 1 million Americans based on a faulty assumption. Congress must intervene Stat News (2026). Available online at: https://www.statnews.com/2026/04/01/ostomy-supplies-competitive-bidding-program-cms/ (Accessed April 27, 2026).

[122] Sengul T Ozakgul A Akyaz DY Aydin Y Karakaya D . The reflections of global climate change on wound and ostomy care: awareness, experiences, and strategies in nursing practices. Int Wound J. (2025) 22:e70729. doi: 10.1111/iwj.70729 40698609 PMC12284731

[123] Garcia Sanchez JJ Chou OHI Davies G Arnold M . Using chronic kidney disease as a model framework to estimate healthcare-related environmental impact. Adv Ther. (2025) 42:348–61. doi: 10.1007/s12325-024-03039-w 39541083 PMC11782431

[124] Jimenez-Lacarra V Martinez-Camara E Saenz-Diez Muro JC Jimenez-Macias E Blanco-Fernandez J . Environmental benefits of reducing patient mobility and hospitalization. Sustainability. (2024) 16:11073. doi: 10.3390/su162411073 30654563

[125] Keil M Viere T Helms K Rogowski W . The impact of switching from single-use to reusable healthcare products: a transparency checklist and systematic review of life-cycle assessments. Eur J Public Health. (2023) 33:56–63. doi: 10.1093/eurpub/ckac174 36433787 PMC9898010

[126] Shoja M Arsalani N Fallahi-Khoshknab M Mohammadi-Shahboulaghi F . The barriers and facilitators to nursing care for patients with permanent colostomy in outpatient centers: a qualitative study. J Educ Health Promot. (2024) 13:72. doi: 10.4103/jehp.jehp_272_23 38559476 PMC10979781

[127] Alansari AN Omri AE Singh K . Staff shortages linked to burnout, depression, and anxiety among outpatient nurses: a cross-sectional analysis at Hamad General Hospital, Qatar. BMC Nurs. (2025) 24:118. doi: 10.1186/s12912-025-03758-7 40859218 PMC12379308

