# Supplementary material for: Where healthcare systems fall short in long-term ostomy care: implications for quality of life, healthcare utilization, and sustainable care pathways
Source: Front Gastroenterol (Lausanne). 2026 Aug 27;5:1920507. doi: 10.3389/fgstr.2026.1920507 (PMC13559913; doi:10.3389/fgstr.2026.1920507)
Supplement: Supplementary file 1 [file Table1.docx]

Supplementary Material

# 1 Supplementary Tables

**Supplementary Table S1**: Database search strategy

| **Database** | **Dates / limits applied** | **Search string** |
| --- | --- | --- |
| **PubMed/MEDLINE** | 2020-01-01 to 2026-12-31; English; Humans | ("ostomy"[MeSH Terms] OR "colostomy"[MeSH Terms] OR "ileostomy"[MeSH Terms] OR "urinary diversion"[MeSH Terms] OR stoma*[Title/Abstract] OR ostomy*[Title/Abstract]) AND ("self-management"[Title/Abstract] OR "self-care"[MeSH Terms] OR "quality of life"[MeSH Terms] OR "patient education as topic"[MeSH Terms]) AND ("health services"[MeSH Terms] OR "patient readmission"[MeSH Terms] OR "health care costs"[MeSH Terms] OR "economic burden"[Title/Abstract] OR telehealth[Title/Abstract] OR "digital health"[Title/Abstract] OR "environmental sustainability"[Title/Abstract]) AND ("2020/01/01"[Date - Publication] : "2026/12/31"[Date - Publication]) AND English[Language] AND humans[MeSH Terms] |
| **Embase (Ovid)** | 2020-2026; English; Human | ('stoma'/exp OR 'colostomy'/exp OR 'ileostomy'/exp OR 'urinary diversion'/exp OR stoma*:ti,ab OR ostomy*:ti,ab) AND ('self care'/exp OR 'quality of life'/exp OR 'patient education'/exp OR 'self-management':ti,ab) AND ('health care utilization'/exp OR 'hospital readmission'/exp OR 'health care cost'/exp OR telehealth:ti,ab OR 'digital health':ti,ab OR 'sustainability':ti,ab) AND [2020-2026]/py AND [english]/lim AND [humans]/lim |
| **Cochrane Library (CENTRAL + CDSR)** | 2020-2026 | (ostomy OR stoma* OR colostomy OR ileostomy OR "urinary diversion"):ti,ab,kw AND ("self-management" OR "self-care" OR "quality of life" OR "patient education"):ti,ab,kw AND ("healthcare utilization" OR readmission OR cost* OR telehealth OR "digital health" OR sustainab*):ti,ab,kw, Publication Year from 2020 to 2026 |
| **CINAHL (EBSCO)** | 2020-2026; English; Human | MH ("Ostomy" OR "Colostomy" OR "Ileostomy" OR "Urinary Diversion") OR TI stoma* OR AB stoma* AND MH ("Self Care" OR "Quality of Life" OR "Patient Education") OR TI "self-management" AND MH ("Health Services Needs and Demand" OR "Patient Readmission" OR "Health Care Costs") OR TI (telehealth OR "digital health" OR sustainab*); limiters: 2020-2026, English, Human |
| **ClinicalTrials.gov** | Study start 2020-2026 | Condition/disease: "ostomy" OR "colostomy" OR "ileostomy" OR "urinary diversion" \| Other terms: "self-management" OR "quality of life" OR "telehealth" OR "patient education" \| Study start date: 01/01/2020 to 12/31/2026 |
| **ICTRP (WHO)** | 2020-2026 | Condition: ostomy OR stoma OR colostomy OR ileostomy \| Intervention: "self-management" OR "patient education" OR telehealth OR "digital health"; date of registration 2020-2026 |
| **Google Scholar** | 2020-2026; first 200 results screened | "ostomy" OR "stoma" OR "colostomy" OR "ileostomy" self-management OR "quality of life" OR "healthcare utilization" OR telehealth OR "environmental sustainability" — sorted by relevance, first 200 records screened per convention for grey-literature engines lacking field-searchable Boolean logic |

**Supplementary sources (hand-searched, not database queries):** International Ostomy Association (IOA), European Ostomy Association (EOA), Ostomy Association of the Americas (OAA), Asian and South Pacific Ostomy Association (ASPOA), and United Ostomy Associations of America (UOAA) websites and publication resources were hand-searched for relevant guidance documents, position statements, and patient-reported data.

**Supplementary Table S2: Literature search and selection flow**

| **Stage** | **Description** | **n** |
| --- | --- | --- |
| Database records identified | PubMed/MEDLINE (412); Embase (587); Cochrane Library (96); CINAHL (268); ClinicalTrials.gov (74); ICTRP (51); Google Scholar (first 200 records screened) | 1,688 |
| Organizational sources | Hand-searched: IOA, EOA, OAA, ASPOA, UOAA | +15 |
| **Total records identified** |  | **1,703** |
| Duplicates removed |  | −612 |
| **Records screened (title/abstract)** |  | **1,091** |
| Excluded at title/abstract screening | Not relevant to review scope | −943 |
| **Full-text records assessed for eligibility** |  | **148** |
| Excluded at full-text review | Pediatric population only (14); short-term/perioperative outcomes only, no long-term follow-up (18); non-peer-reviewed commentary or case report without systematic data (9); duplicate cohort/population reported elsewhere (7); full text unavailable (4); outcome outside review domains (4) | −56 |
| **Studies included in narrative synthesis** |  | **92** |
